# Cooperation among Wirelessly Connected Static and Mobile Sensor Nodes for Surveillance Applications

**DOI:** 10.3390/s131012903

**Published:** 2013-09-25

**Authors:** Edison Pignaton de Freitas, Tales Heimfarth, Alexey Vinel, Flávio Rech Wagner, Carlos Eduardo Pereira, Tony Larsson

**Affiliations:** 1 Department of Information Technology, Federal University of Santa Maria in Frederico Westphalen, CESNORS, Frederico Westphalen 98400-000, Brazil; 2 Electrical Engineering Department, University of Brasília, CP 4386, Brasília 70910-900, Brazil; 3 Department of Computer Science, Federal University of Lavras, CP 3037, Lavras 37200-000, Brazil; E-Mail: tales@dcc.ufla.br; 4 School of Information Science, Computer and Electrical Engineering, Halmstad University, PO Box 823, Halmstad 301 18, Sweden; E-Mails: alexey.vinel@hh.se (A.V.); tony.larsson@hh.se (T.L.); 5 Electrical Engineering Department and Institute of Informatics, Federal University of Rio Grande do Sul, CP 15064, Porto Alegre 91501-970, Brazil; E-Mails: flavio@inf.ufrgs.br (F.R.W.); cpereira@ece.ufrgs.br (C.E.P.)

**Keywords:** wireless sensor network coordination, bio-inspired networking, mobile sensor nodes, surveillance systems

## Abstract

This paper presents a bio-inspired networking strategy to support the cooperation between static sensors on the ground and mobile sensors in the air to perform surveillance missions in large areas. The goal of the proposal is to provide low overhead in the communication among sensor nodes, while allocating the mobile sensors to perform sensing activities requested by the static ones. Simulations have shown that the strategy is efficient in maintaining low overhead and achieving the desired coordination.

## 1. Introduction

The integrated use of mobile and static sensor nodes is a promising approach that allows the deployment of *advanced surveillance systems* [1,2]. On one hand, simple and cheap (low-end) static ground sensor nodes allow massive deployment, making it possible to cover large areas [3]. On the other hand, mounting more expensive and more sophisticated (high-end) sensors on mobile platforms [4,5] allows their usage in different locations of a covered area [6]. Combining these two types of sensors, the low-end sensors can trigger the usage of the high-end ones in different locations upon demand, which allows having a lower number of these expensive sensors, thus resulting in a positive impact in the overall cost of the system, without losing functionality.

In respect to the mobility feature, despite the usefulness of bi-dimensional mobile platforms on the ground, such as *Unmanned Ground Vehicles* (*UGVs*), the sensor's operational area may be reduced due to either the terrain's geography or other obstacles [7]. Three or two dimensional mobile platforms in the air (two by moving at a fixed height), such as *Unmanned Aerial Vehicles* (*UAVs*) [8], may provide better results as they have the ability to freely move the sensor to the desired locations, avoiding obstacles. Small UAVs, such as those presented by MLB Spyplanes [9], are much cheaper than large UAV platforms, such as Predator and Globalhawk [10] and are able to perform missions in regions which large platforms would not be able to access, such as urban environments [11,12]. Based on these arguments, the interest for small UAV platforms to act as the mobile sensors is reasonably rational.

In order to make this above discussed combined WSN setup efficiently work, this paper presents a networking strategy to allow the cooperation of low-end static sensors on the ground and high-end mobile sensors in the air, applied to the scenario of large areas surveillance. The proposed strategy aims at providing efficient communication among the sensor nodes, reducing the number of messages exchanged in the network. The goal of this reduction is to decrease the overhead in terms of resource consumption of the low-end static sensor nodes, as communication is known as a resource intense task in WSNs and an important concern for these sensor nodes [13]. In other words, we propose a method to deliver alarm messages issued by the static sensor nodes to the UAVs, so that the desired cooperation needed to perform resource efficient area surveillance is achieved. To address this alarm messages delivery problem, the approach proposed in this work uses a decentralized strategy, based on artificial *pheromones*, inspired by the biological mechanisms used by ants to track food in the nature [14]. Artificial pheromones have previously been applied to distributed coordination by means of stigmergy, the indirect communication using environment cues [15]. Pheromone marks are deposited in the environment forming a trail while biological entities such as ants are moving. The pheromone provides information to other entities when they pass over it. Artificial pheromones have the same feature, *i.e.*, to provide information, as well as the same behavior; they also lose their strength over time, modelling the evaporation of the real pheromones. These are the main characteristics explored in the proposed approach.

The *main contributions* of this paper can be summarized as follows:

1. A detailed model of WSN composed of static low-end ground sensor nodes and small UAVs applied to a scenario of large area surveillance is proposed;
2. Reusing a known concept of pheromone-based communication for the studied scenario, we propose a novel protocol for the alarm message delivery from static to mobile sensor nodes, including three different mechanisms: trail-follow and trail-search as well as pheromone distribution over the ground sensors. The innovative aspect of this approach is supported by the way the stigmergy concept is used to guide the communication and coordinate the sensor nodes. In contrast to previous works, the proposed approach does not have route discovering phases to allow the communication among the nodes. Moreover, it considers arbitrary trajectories of the mobile nodes instead of pre-know information about them.

Intensive simulation study of the proposed protocol is performed, and its overhead is evaluated against two reference approaches, namely: (a) an optimal one, which takes advantage of the global system state knowledge to minimize the needed transmissions; and (b) a flooding one, which provides maximal possible overhead due to the naive retransmission of the alarm by all the sensors.

The remainder of the paper is organized as follows: Section 2 discusses related works in the area. Section 3 presents the target application and the studied scenario. Section 4 describes the proposed bio-inspired networking strategy to perform the cooperation among mobile and static sensor nodes, while Section 5 presents and discusses experimental results. Finally, Section 6 concludes the paper with final remarks and directions for future work.

## 2. Related Works

The AWARE project [1] aims at integrating a sensor network of resource constrained ground nodes with mobile sensors, carried on the ground by UGVs and in the air by UAVs. In a large sense this work is closely related to ours and an idea that is shared in both is the combined use of ground sensors and UAVs taking part in the same sensor network and cooperating to achieve surveillance mission goals. In [16] a rendezvous-based data dissemination protocol to deliver critical alarm messages issued by the static sensor nodes to the mobile ones in the context of AWARE project is presented. This approach divides the network of static sensors in a set of hexagonal cells, like a honeycomb, which is a solution used in other applications such as cellular phones. Both alarm messages from the static sensor nodes and queries from the mobile ones are forwarded to central parts of the network following the hexagonal organization of the nodes, and then forwarded to their destinations. Our approach is more flexible because the nodes do not need to keep information about their neighbors, as it is needed in clustered organizations such as the proposed hexagonal one in AWARE. Moreover, in our approach there is no preferable region of the network to where the messages should be forwarded, such as the centre of the hexagons in their work.

In [17], an approach exploring the concept of scent marking used by primates for territorial demarcation is proposed to provide cooperation among mobile sensors, by using static sensors and Radio Frequency Identification (RFID) tags. On one hand, this work is closely related to ours because its concept of scent marking is very similar to the stigmergy approach behind the pheromones used in our work. In their proposal, mobile sensors are robots that move around an area, and when they find events of interest in a given region they leave RFID tags or sensors covering this region. If they need help to complete a given task the information stored in the tags will drive other robots towards the region where their help is required. The main difference from our work is that they deploy new nodes in the network, in an *ad hoc* fashion, as an attempt to disseminate the information that must be received by the other mobile nodes, in this case a help request. On the other hand, our approach assumes an already deployed network of static nodes, which may perform a number of sensing missions, with which the mobile ones cooperate. In this sense, we try to use the resources available in the network in a rational way so as to not waste them, while the proposal in [17] relies on additional resources being added to the network according to the needs. In [18], a biologically inspired approach for division of labour in Sensor/Actuator Networks (SANETs) is proposed. They also explore the ant pheromone mechanism, in a scenario closer to what is discussed in [17], but focusing on a statistical method to perform route decisions. In order to perform these decisions, the proposed networking mechanism is based on information obtained during a route discovery phase, which is an important difference in relation to our work. In our proposal the route is discovered by the message that has to be delivered to the destination node, avoiding the overhead of a precedent route discovery phase, as proposed in [18].

A proposal to solve the problem of message dissemination in a multi-level WSN composed by static and mobile sensors on the ground and UAVs moving in the air is presented in [19]. Their approach is based on the epidemic routing concept, but uses a probabilistic decision to forward or not incoming messages, so that a more efficient usage of the communication medium is achieved. As the nodes on the ground are also able to move, and considering the urban scenario addressed in their work, nodes may have different numbers of neighbors during system runtime. Therefore, they investigate the adaptation of the forwarding decision process according to the nodes' neighborhood, by a proposal called Adaptive Probabilistic Epidemic Protocol (APEP). An aspect that is common with our work is the need to deliver messages from the ground sensors to the UAVs. As the positions of the UAVs are unknown, they try to address the problem by a flooding-based method, whose overhead is reduced by the probabilistic decision making process and by the knowledge about the neighborhood of a node on the ground. Our approach is different because it tries to provide information about the UAVs' movement to the ground nodes, so that the ground nodes can forward messages in the direction of the most suitable UAVs, thus reducing the drawbacks typical of flooding-based mechanisms.

In [2], a divide and conquer solution for surveillance of large areas is proposed, in which static and mobile sensors provide coverage in an area. Static sensors are deployed in selected regions, such as the boundaries of the area, while mobile sensors move around these regions. The coverage problem is also studied in [20], in which the capability of area coverage by regularly and randomly deployed sensors is compared. The main difference to our approach is that we focus on the cooperation among different types of nodes, while they analyze the nodes' distribution in order to increase the area coverage. Focusing in this aspect, their approach has no commitment in reducing communication costs as presented in our proposal, even considering that specific nodes' arrangements can provide this feature as a side effect.

Other related approaches [21–24] handle the problem of mobile sinks in WSNs, which can be mapped to the scenario addressed in our work, considering the UAVs as mobile sinks. The goal of delivering sensed data to mobile sinks reported in these related works is comparable to our concept of alarm delivery. Some of these proposals consider that the mobile sinks may decide about their movement in order to facilitate the message delivery by static sensor nodes and thus optimize the energy usage in the network as a whole [21,22]. Other proposals assume that the movement of the sinks is at least predictable, as in [23]. The assumptions about mobility presented in these works differ from ours, as there is no predictability in the movement pattern of the UAVs in our work. Moreover, the UAVs cannot present a preference to move towards the direction of a given group of nodes, before the occurrence of an alarm, as there is no indication that they would be needed in that location. Also considering mobile sinks, in [24] the concept of delay tolerant networks (DTN) is explored. Authors report experiments with the deployment of mobile nodes to collect information from static sensors to be used in agricultural applications. To drive the movement of data from the sensor nodes towards a central processing node via the mobile sink, authors use potential-based routing that sets higher potential to data providers, an intermediary potential to the mobile sinks and a lower one to the central node. Their potential-based routing is to some extent comparable to our pheromone-based one, but, while the pheromone-based strategy addresses the dynamicity imposed by the movement of the UAVs, their potential-based one is statically configured before runtime.

In [25] the deployment of additional mobile nodes that are used as relay nodes to improve the network performance is proposed. According to the network lifetime dynamics, *i.e.*, nodes' movement, interferences, among other events, these additional relay nodes change their placement according to the link quality, so that network connectivity is enhanced. This work explores an approach based on deployment of additional mobile nodes, and of controlling these nodes' movements to facilitate message delivery, combining the ideas presented in [17,21] and [22], thus having the same comparison to our proposal as discussed above.

In summary, the related works can be classified in two categories: (a) works that try to solve a similar problem, but present a different approach or modeling assumption; and (b) works that consider a similar system, but focus on different problems. It is important to highlight the differences (advantages) of our proposal in relation to the analyzed works in the first category. An important advantage of our proposal in relation to those described above is that it does not have any dependency on predefined conditions to work, such as the predefined preferred locations to send alarm messages in AWARE [16] or specific movement patterns of the mobile nodes as in [22,23].

## 3. Application Scenario and System Model

### 3.1. Application Scenario

The scenario studied considers surveillance systems using two different types of sensors: static on the ground and mobile in the air (UAV-carried or simply UAV). There is a number of static ground sensor nodes spread in the area of interest, called mission area, according to a given distribution, which can be random or following a predefined pattern. The ground sensor nodes perform simple measurements, such as differences in magnetic field and CO_2_ concentrations, among others, which can indicate the presence of objects or occurrence of events of interest, such as vehicles or people, or the occurrence of a fire. Some UAVs fly over the mission area, following a random or predefined movement pattern. UAVs are equipped with more sophisticated sensors, such as visible light cameras, infrared cameras, and synthetic or inverse synthetic aperture radars. The UAV sensors typically provide more detailed information compared to the static sensors on the ground. The number of UAVs is however much lower than the ground sensor nodes, and the idea is to make them work cooperatively, so that they complement each other. The distribution of static ground sensor nodes is assumed to ensure that they cover the whole area and, when they identify a possible event of interest, they trigger an alarm that is sent to the UAVs, equipped with sophisticated sensors, to perform more accurate observations.

The sensor nodes communicate with each other via wireless links within a tunable, but limited, communication range. Due to the broadcast nature of wireless media, all nodes in the range of a sending node receive the sent messages.

The system behavior is defined as follows. Ground sensor nodes are configured to detect phenomena indicating possible threats, which are defined by a set of threshold levels of their measurements. When the acquired measurements reach a configured threshold level, a “match” with the detecting criterion is achieved. In the occurrence of a match, the sensor node issues an *alarm*, which is received by all nodes that are within its communication range.

Alarm messages are sent as single communication packets containing a timestamp, the position of the issuer node, and the type of the possible threat. The first two components of the alarm message enable unique identification, avoiding alarm duplication, assuming the atomicity of the events reported by the alarms, which means that each alarm that is sent indicates a different threat. Consequently, if the indicated threat is a group of persons or vehicles, they are handled as a single threat. This assumes that neighboring nodes on the ground cooperate to aggregate information needed to characterize threats before issuing an alarm.

**Figure 1.**
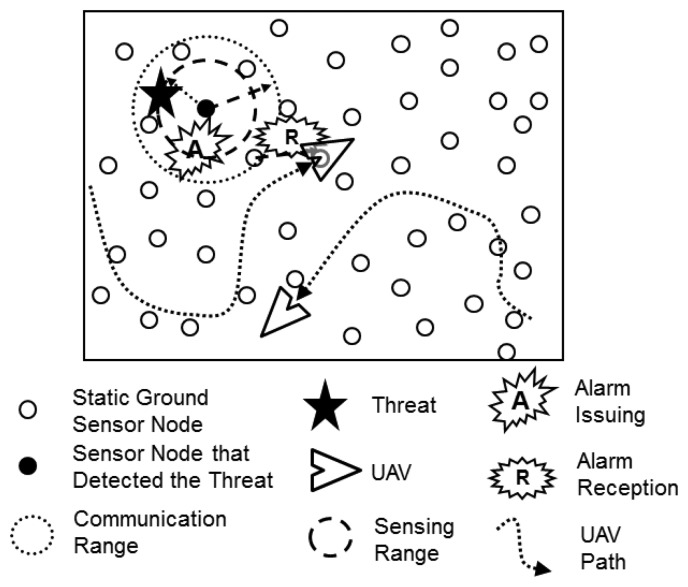
Overview of the application scenario illustrating threat detection, alarm issuing and alarm reception.

The main elements of the described scenario are presented in Figure 1. In the figure it is possible to observe the detection of a possible threat by a ground sensor node. This node issues an alarm that is received by all its neighbor nodes. One of these neighbors relays the alarm, which is then received by the neighbor static sensor nodes and by a nearby UAV.

At the occurrence of an alarm, one UAV, equipped with more sophisticated sensors, takes the responsibility for the alarm and flies towards the area where this alarm was issued, in order to gather further information about the possible threat and confirm it as a real threat, e.g., an intruder or a fire spot.

### 3.2. System Model

The assumed model of each of the above mentioned elements that compose the scenario is presented in the sequence.

#### 3.2.1. Operation Area

The considered scenario is composed by a rectangular area in which each element (threats and sensors) is identified by its Cartesian coordinates, *x* and *y*. The area may be subdivided in sub-areas, consisting of a sub-set of points in the area, which may have properties assigned to them, such as weather conditions for a specific sub-area.

#### 3.2.2. Threat Model

A threat appearance means its detection by a sensor node. The threats may be static or mobile, depending on the application semantics. If mobile, they are considered to move with a constant speed, but different threats may have different speeds. Mobile threats may also randomly change the direction of their movement. The appearance of threats is defined by either a given arrival probabilistic model or a deterministic model describing specific conditions of appearance.

#### 3.2.3. Static Sensor Model

The positions of the static sensor nodes are given by their corresponding coordinates, and this position is assumed to have been established during system deployment and does not change during system operation. It is also assumed that static sensor nodes know their own position, which is possible by means of a Global Positioning System (GPS) [26] device or any other positioning mechanism, such as algorithmic solutions [27]. These nodes have their communication capabilities defined by a communication range *r_c_*.

**Figure 2.**
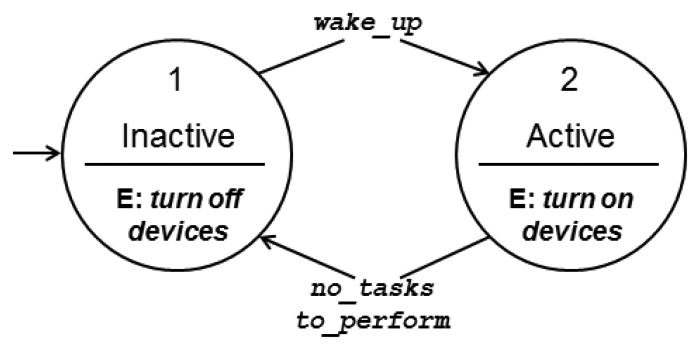
Finite state machine for ground sensor nodes.

The operation of static ground sensor nodes is based on an energy saving model [28], in which the nodes sleep most of the time, which means that they turn off all or almost all their devices. A duty cycle mechanism defines the periods in which the nodes wake up (represented by *t_w_*), and stay awake for a time window *t_a_* during which the nodes perform their activities, *i.e.*, they turn on their processing, communication and sensor devices to process information, sample the environment and exchange messages with other nodes. Figure 2 presents the finite state machine (FSM) describing the ground sensor nodes' behavior, with the two possible states, active and inactive (sleep), and the transitions between them.

#### 3.2.4. Mobile Sensor Model (UAV)

The UAV instance is considered to have an internal state at any moment of time, which contains two components: a physical state and an engagement state:

(a) *Physical State (PS)*: this state includes information on the UAV's current position, speed, heading angle (*ψ*), communication range (*R_com_*), and remaining energy resources.
(b) *Engagement State (ES)*: according to the detected threats in the surveillance area and to the respective alarms issued, a UAV can be in one of the following states: *Idle*, *Engaged* or *Busy*. The first state may occur when a UAV is just patrolling the area, being considered idle and ready to engage in performing a task over a threat informed about by an alarm. The second state occurs when a UAV is engaged in performing a task related to a threat, but it is not performing it yet, e.g., it is still moving towards the location where the threat was detected. The third state occurs when a UAV arrives at the threat location and starts handling the threat, *i.e.*, it is performing a task over it, for example tracking it or just confirming it as a threat.

Received alarms are stored in a queue and then handled according to a first come first serve policy.

**Figure 3.**
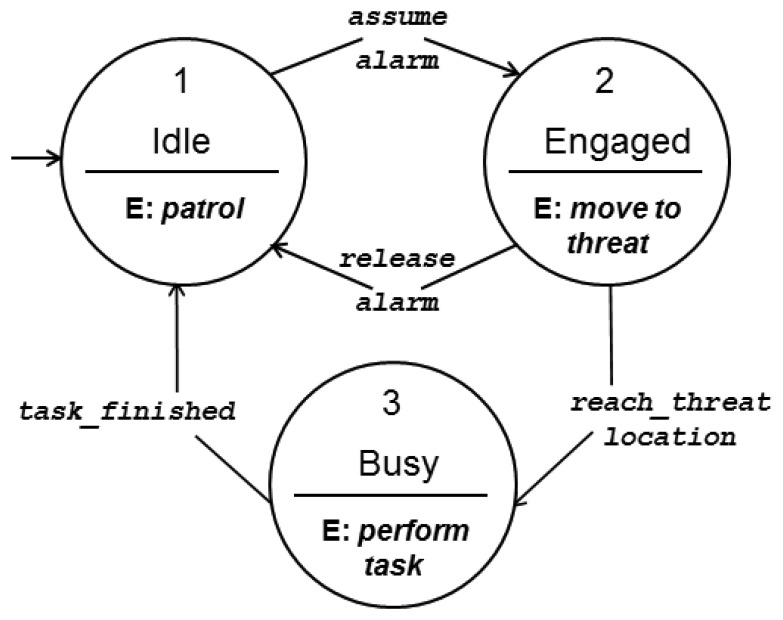
Finite state machine for unmanned aerial vehicles.

Figure 3 presents the FSM for the UAVs, in which one can observe the possible states and transitions among them. The transition from state 1 (Idle) to state 2 (Engaged) happens when a UAV receives an alarm and assumes the responsibility for handling it.

Once an engaged UAV reaches the threat location, after being informed about it by an alarm, it transits to state 3 (Busy). When it finishes handling the threat it transits to the Idle state, unless it is engaged also with any other alarm and instead transits back to 2 (Engaged).

**Figure 4.**
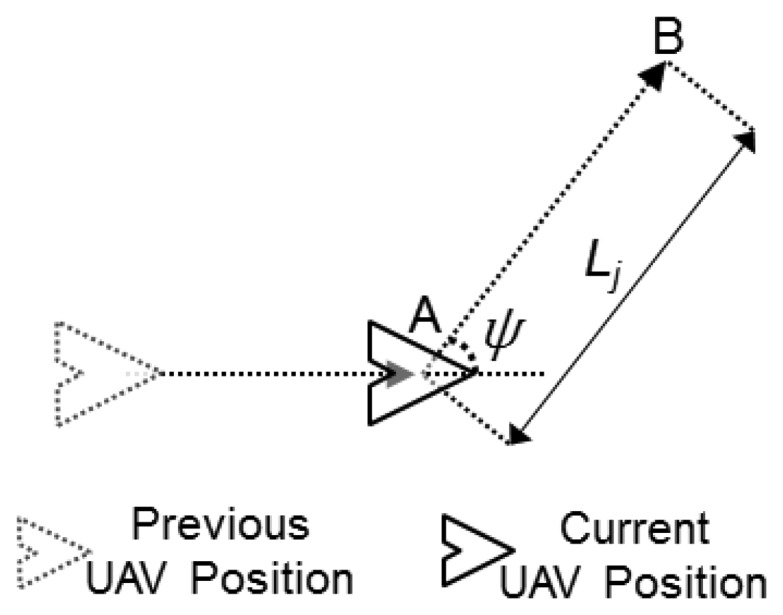
Unmanned aerial vehicles' movement step and direction.

The adopted kinematic model considers that the UAVs move along continuous trajectories with constant speed and with a constrained turning angle [29]. This turning angle may vary after the UAVs fly a given distance, or movement step *L_j_*, according to the adopted movement pattern. Figure 4 presents an example in which a UAV turns at point *A*, to move a distance *L_j_* towards the defined direction until it reaches point *B*, where it will change its direction again.

## 4. Pheromone-Based Alarm Delivery Concept

In this work, the use of pheromones is proposed to guide the alarm messages issued by a ground sensor node through the network until the alarm delivery to a UAV. When a threat is detected and an alarm is issued, the alarm tries to reach the closest UAV. This is performed by routing the alarm via the static sensor nodes that store the strongest pheromone marks over the area. Then, when the alarm reaches a sensor node that has a UAV in its communication range, the alarm is delivered to this UAV, which will eventually move to the area where the alarm was generated. This strategy is further referred to as heuristic-P.

### 4.1. Trail-Follow Mechanism

Following the above outlined principles, UAVs that are not handling any threat (they are either idle or engaged) leave pheromone marks over the sensors in the area which they cross, by means of broadcast beacon messages. These pheromone marks are collected by the ground sensor nodes that are deployed in the area through which the UAVs have passed. When a threat is detected by a ground sensor node, it issues an alarm that is routed through the network as already mentioned. Following this principle, alarm messages can be compared to follower ants that follow the trails left by other ants that found food in the nature (the UAVs in this case). Heuristic-P is inspired in [30], which presents a pheromone-based strategy for migration of services in a WSN. In [30], the pheromone concentration determines the places where the services are required. In heuristic-P, instead of services, alarms move through the network following the pheromone trails to reach UAVs.

**Figure 5.**
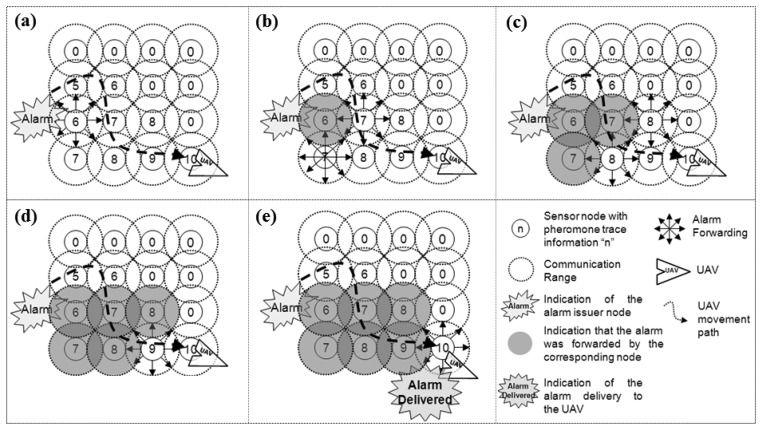
Trail-follow mechanism example used in the pheromone-based alarm delivery step by step from the alarm issuing towards its delivery: (**a**) Alarm issue; (**b**) Alarm propagation closer the alarm issuer node; (**c**) Alarm propagation proceeding; (**d**) Alarm propagation getting closer to the nodes in the range of the UAV; (**e**) Alarm delivery.

Figure 5 illustrates an example of how an alarm issued by a sensor node (Figure 5a) is routed through the network, following the pheromone trail (Figure 5a–d), until it reaches and is delivered to a UAV (Figure 5e). This mechanism is called *trail-follow*.

The pheromone marks in the nodes are illustrated in Figure 5 by numbers placed in the centre of the circles representing ground sensor nodes. The higher the number is, the stronger the pheromone mark is. In this example, 10 represents the highest pheromone level, which represents a situation in which a sensor node just received a beacon from a UAV that is flying over it, while number 0 represents the opposite situation, in which the sensor node has no pheromone mark. Notice that during the trail-follow, the alarm can be redundantly sent by more than one node, according to the pheromone concentrations.

### 4.2. Trail-Search Mechanism

**Figure 6.**
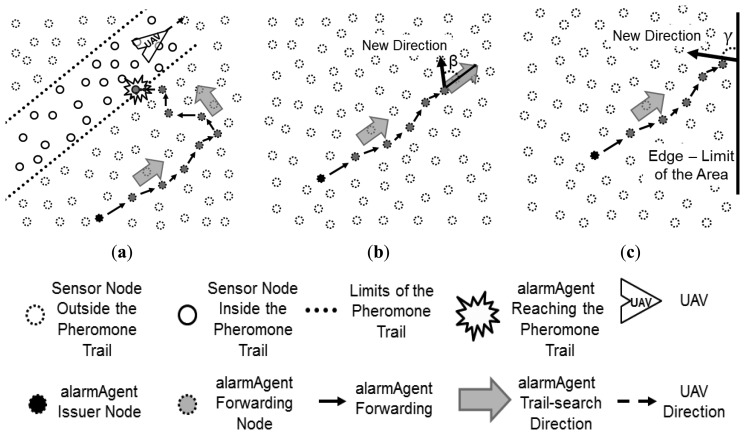
Trail-search mechanism: (**a**) Illustration of the mechanism concept, showing the direction followed by the alarm from the alarm issuer node until reaching a trail, in which the followed direction is changed once; (**b**) General case for forwarding direction change; (**c**) Particular case for forwarding direction change in the limits of the area.

In case an alarm is issued by a node that has no pheromone trace (“0” pheromone mark on it), a direction is randomly chosen and the alarm follows this direction until it finds a pheromone trail. When a pheromone mark is found in a node, it follows the respective trail as explained above. This situation is more likely to occur during system initialization, and in cases in which the number of UAVs deployed in the system is very low and/or the size of the trail is small in relation to the size of the mission area. This mechanism is called *trail-search*, which is illustrated in Figure 6.

As can be observed in Figure 6a, while performing the trail search, an alarm follows the nodes towards a given direction, which is randomly chosen from the position of the alarm issuer node, until it reaches a trail or satisfies a given condition to change the direction of its forwarding. This condition can be defined as a number of hops or just by reaching the limits of the mission area, for instance.

Considering the direction which the alarm follows in the trail-search, in the case in which the decision to change the forwarding direction is taken after a given number of hops, when arriving at a node that fulfils this condition, a new direction is defined as an angle β. This angle is randomly chosen according to a uniform distribution in the interval (−π/2, π/2) in relation to the current direction, as depicted in Figure 6b. If the alarm reaches a node that is located close to an edge that limits the operation area, a new direction in relation to this edge is defined as an angle γ randomly chosen according to a uniform distribution in the interval (0, π), as shown in Figure 6c. Notice that in the first case, in which the condition to change direction is determined by a number of hops, if the alarm does not find a trail and reaches one of the edges that limits the area, the same behavior presented in Figure 6c is taken.

The implementation of the trail-search mechanism can be done by using a simple greedy position-based routing mechanism [31]. The one adopted in this work considers the selection of the forwarding node based on the angle that the line that links the current node to a neighbour node forms with the reference direction. The node that has an angle closer to β is selected to proceed with the alarm forwarding process. When an alarm reaches a node that is placed in a position closer than one communication range from one of the edges that limit the operation area, it is considered that the alarm reached a limit edge, and thus a new angle is chosen, as seen in Figure 6c.

The trail-search mechanism is also performed in cases in which the network is disconnected and the alarm was performing a trail-follow, but it reaches a limit of a partition of the network and is not able to proceed following the trail in the right direction. Then it starts a trail-search in an attempt to find another trail. While the alarm is performing the trail-search towards a given direction and then reaches a situation in which it gets stuck due to network disconnection, it will select a new direction to proceed with the trail-search. This behavior is similar to the behavior of an alarm that reaches a limiting edge of the operation area.

The pheromone marks stored by the ground sensor nodes have two components: a temporal and a spatial one. The first component defines the elapsed time since the UAV beacon was received by the ground sensor node, while the second one defines the distance between the UAV and the sensor node, which can be achieved by different methods, such as the received signal strength indication (RSSI) of the incoming beacon or by the current UAV's GPS position sent in the payload of the beacon message, for instance. These two components are used to define the pheromone concentration *C_p_*(*t*), which decays with the elapsed time since a ground sensor node receives a beacon from a UAV, defined as *C_p_*(*t*) = *C_p_* (*t* – 1)*r*, where *r* is the tunable pheromone decay rate. This decay rate may have a predefined fixed value if all UAVs are assumed to have the same and constant speed; otherwise they transmit this decay rate to the ground sensor nodes in the payload of their beacon messages.

### 4.3. Pheromone Distribution over the Ground Sensor Nodes

The example presented in Figure 5 shows a regular distance distribution of the ground sensor nodes. This simplifies the alarm forwarding process, as the pheromone information is evenly distributed among them, assuming for instance that the received signal strength indication (RSSI) is used to define the pheromone level.

**Figure 7.**
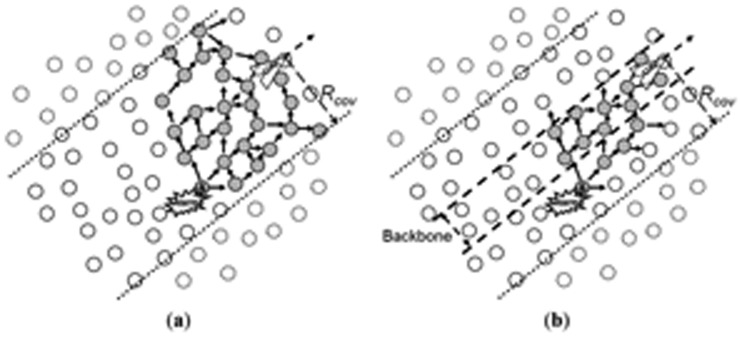
Alarm forwarding inside the pheromone trail: (**a**) Trail-follow without backbone; (**b**) Trail-follow with backbone, showing that a smaller number of nodes are involved in the alarm message forwarding.

However, considering a more general case, in which the sensor nodes are placed using a uniform random distribution on the ground, such a regular distance distribution of the pheromone information would hardly be achieved, which may cause problems in the alarm forwarding. The main problem then is the increased number of sensor nodes that would forward a given alarm in the trail-follow process, thus unnecessarily increasing the number of sent messages, leading to a waste of energy resources. To tackle this problem, a special region is defined in the center of the pheromone trail, which constrains the broadcast of alarm messages sent towards the UAV. To understand the reason for this, Figure 7a shows how an alarm propagates in the trail without the definition of such region.

As shown in Figure 7a, the alarm is broadcasted towards the direction of the movement of the UAV, by flooding the nodes towards the UAV. Notice that this generates a number of redundant forwarding alarm messages that are unnecessary. Using the spatial component of the pheromone stored by the ground sensor nodes, it is possible to restrict the alarm forwarding to the nodes closer to the real path followed by the UAV. Like this, the alarm would be forwarded to the inner part of the trail, which is called the trail backbone, and, by reaching this inner part, the trail-follow can be constrained by its limits. The width of the backbone can be defined in terms of the UAVs' communication coverage range on the ground (*R_cov_*), and it can be wider or narrower according to the accepted level of redundancy. The reduction in the number of messages when using this backbone concept can be seen in Figure 7b.

**Figure 8.**
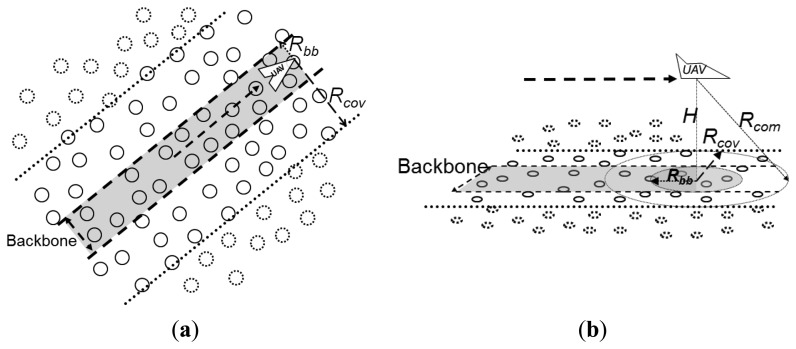
Pheromone trail with backbone: (**a**) Top-down 2 dimensional view; (**b**) 3 dimensional view.

Figure 8 visualizes the proposed backbone from a top-down two-dimensional perspective (Figure 8a) similar to Figure 7 and from a three-dimensional perspective (Figure 8b). In the figure it is possible to observe the UAV's communication range *R_com_*, its coverage on the ground *R_cov_*, and the delimitation of the backbone coverage *R_bb_*.

Alarms issued by nodes located inside the backbone follow the backbone until they deliver their alarms to the corresponding UAV. Alarms issued by nodes outside the backbone are first forwarded towards the backbone and then follow the backbone, as in the example of Figure 7b. This approach bounds the flooding of messages used to forward alarms, *i.e.*, the overhead of the trail-follow mechanism, to the limits of the backbone. This avoids the retransmission of alarms by a large number of nodes, as it would be the case when a backbone is not adopted, as illustrated in Figure 7a. In a limit case, the backbone could be defined to be narrow enough such that trail-follow mechanism would not represent a controlled flooding inside a bounded region, but a direct transmission node by node towards the UAV. In this case, on one hand the overhead of the trail-follow mechanism would achieve its minimum value. On the other hand, the mechanism would not present redundancy, thus it is possible to state a tradeoff between these too concerns, namely the overhead and the provided redundancy.

## 5. Experiments and Results

Results from simulations of the described bio-inspired approach are presented for different setups to assess its efficiency. Basic simulation parameters holding for most setups are first presented, while parameter variations used to assess specific aspects are presented along the subsections according to the specific setups. To perform the simulations, a tool called GrubiX was used. GrubiX is a redesign and extension of the ShoX *ad hoc* network simulator [32], which is being evolved by the addition of new features, such as the possibility to have nodes with different characteristics (e.g., mobility and energy models) in the same simulation. GrubiX has also got improved reuse mechanisms in relation to those provided by ShoX, by an architecture with minimized core functionalities that provides more flexibility to the development of new simulations.

### 5.1. Basic Simulation Parameters

Based on the scenario presented in Section 3, the modeled and simulated environment was defined as a squared area in which static ground sensor nodes are randomly deployed with independent uniform probability (homogeneous Poisson point process in two dimensions, which generates a geometrical random graph). The sensor nodes are set to have a communication range of *r_c_* = 350 m and deployed with a density of 50 nodes/km^2^ for all area sizes tested, providing a connected network according to the conditions discussed in [33]. This is an important assumption, as gaps in the network connectivity negatively impact the results, since these gaps may stop the alarm forwarding process.

The trail-search mechanism is set to choose a random direction (0, 2π) according to a horizontal reference from the alarm issuer nodes, and to change its direction when an alarm reaches the edges of the mission area, choosing an angle in the interval (0, π) in relation to the edge.

UAVs in the model scenario patrol the area by flying at an altitude of 250 m according to a random waypoint movement pattern following the model presented in Figure 4, with a movement step length *L_j_* equal to 500 m and a heading angle ψ randomly chosen in (0, 2π), and at 100 km/h speed. Their communication range *R_com_* is 500 m. The choice of these setup parameters is based on the characteristics of the scenario analyzed in this work, considering the usage of small (Mini or Micro) UAVs that have operational range of 10 km [34], using COTS communication technologies such as those based on IEEE 802.15.4 (extended range version) radios. Unless stated differently, the trail-follow mechanism uses a backbone range *R_bb_* equal to half of the UAVs' communication coverage range on the ground *R_cov_*. The pheromone decay rate *r*, is established to 0.995 units per second, and, after 180 s, the sensor nodes consider that the pheromone mark disappears, *i.e.*, its level is set to 0. The described parameters provide trails with width close to 1km and maximum length of 5 km, which is reached when the UAV flies in straight line for a period equal to the trail lifetime (180 s). The main simulation parameters are summarized in Table 1.

The simulations were divided into different sets, according to variations that allow the assessment of different features and considering a corresponding goal for each set. Additionally, 100 runs were performed for each set.

**Figure 9.**
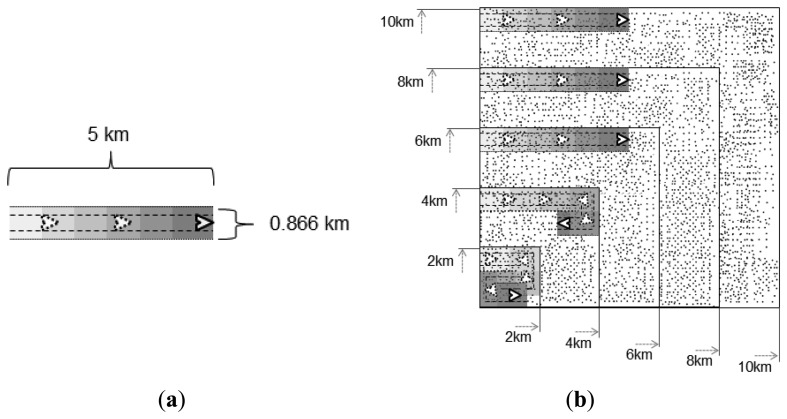
(**a**) Representation of the area that a trail can cover; (**b**) Areas used as test case scenarios for simulations with the representation of how much can be covered by a trail.

Squared areas with five different dimensions were used as test scenarios for the simulations, namely 2 km × 2 km, 4 km × 4 km, 6 km × 6 km, 8 km × 8 km, and 10 km × 10 km. Figure 9 presents those areas in scale for comparison reasons.

Each area presented in Figure 9a illustrates a pheromone trail in scale with dimensions described by the above parameters used for the simulations, while Figure 9b presents the areas used as test case scenarios, providing a visual perspective of how much of each area can be covered at most by a trail of one UAV, when it is flying in a straight line. The gray gradient of the trails in Figure 9a (and the corresponding parts of Figure 9b) represents the strength of the pheromone level stored by the ground sensor nodes in the corresponding positions, in which the lighter the gray is, the weaker the pheromone level is.

### 5.2. Assessment of the Proposed Pheromone-Based Approach

The first set of simulations is aimed to assess the usefulness and efficiency of the basic pheromone strategy (heuristic-P) with the backbone in the trails to deliver alarms to the UAVs. Different numbers of UAVs and threats are considered.

The metric used in the evaluation is related to the cost associated with the alarm delivery, which is calculated in terms of the total number of messages that were sent during each simulation run to deliver the alarms to the UAVs. The results presented are average values based on hundred simulation runs for each average, and the heuristic-P method is compared with both an optimal and a flooding-based solution.

The optimal solution is assumed to implement the minimum number of hops from the alarm issuer node to the closest UAV, thus minimizing the cost in terms of the number of messages that are transmitted in the network to deliver an alarm, and then it is called Optimum-C. The simulated model of such an optimal solution uses knowledge about the global state of the simulations, which gives access to the positions of all sensor nodes and UAVs, such as to choose the shortest path from the alarm issuer node to the closest UAV. In practice, the implementation of an equivalent solution would imply a very large communication overhead, as each time the movement of a UAV changes its connections to the ground sensor nodes, a flooding to the whole network would be needed to update the new routes in all the sensor nodes. The flooding-based solution considers that an alarm is forwarded by sensor nodes in all directions from the position of the alarm issuer node until the alarm reaches a UAV.

A square area of 4 km × 4 km was considered as scenario for this first set of simulations. The scenario was simulated with the combinations of three numbers of UAVs (1, 2, and 4), and four different numbers of threats (1, 3, 5, and 7) appearing in the area according to a uniform random distribution within a time window of 180 seconds, which is the pheromone trail lifetime established by the parameters presented above. The rationale for this specific arrival model is to analyze the disturbances that this increasing number of threats may induce in the achieved results in relation to the pheromone trail lifetime. Consistently with this goal, the specific numbers of threats were chosen to stress these possible effects according to the tested numbers of UAVs.

**Figure 10.**
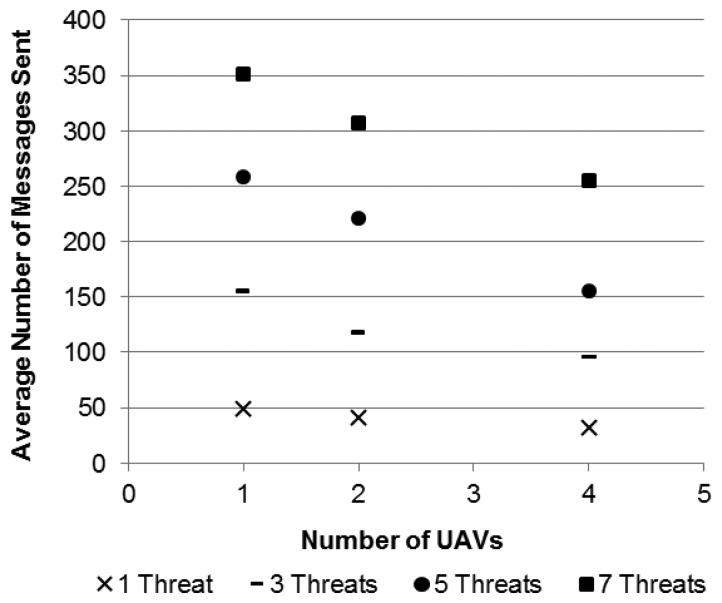
Comparison among the usage of different numbers of Unmanned Aerial Vehicles in terms of number of messages sent for the different setups.

Table 2 presents the statistics for the number of messages sent per UAV in this scenario. It is possible to observe the large difference in results provided by an ordinary flooding-based solution, the proposed pheromone-based one, and the optimal reference solution. It is also possible to observe that the proposed solution presents a fairly good efficiency if compared to the optimal one. Besides, the proposed solution scales with the increasing number of appearing threats, as it is graphically shown in Figure 10. Results in Figure 10 indicate that the increase in the number of employed UAVs reduces the amount of messages sent by a rate of 20% in average in relation to the closer setup with fewer UAVs, *i.e.*, this reduction is seen when the setup with two UAVs is compared to the one with onw UAV and when the setup with four UAVs is compared to the one with two UAVs.

### 5.3. Scalability Analysis

To assess the scalability of the proposed solution, a second set of simulations was performed for each number of UAVs (1, 2, and 4), in which areas with increasing sizes were considered in addition to the one used as scenario for the first set (4 km × 4 km), namely: 2 km × 2 km, 6 km × 6 km, 8 km × 8 km, and 10 km × 10 km, as illustrated in Figure 9b. The assessment was done for the same number of threats (1, 3, 5, and 7) and following the same arrival process described above. The presented results are averages for the group of 100 simulation runs, as the ones presented before for the first simulation set. The scalability analysis is based on the cost metric. In order to better evaluate the proposed method, the results for this metric were divided according to the specific parts of the overall strategy (trail-search and trail-follow).

**Figure 11.**
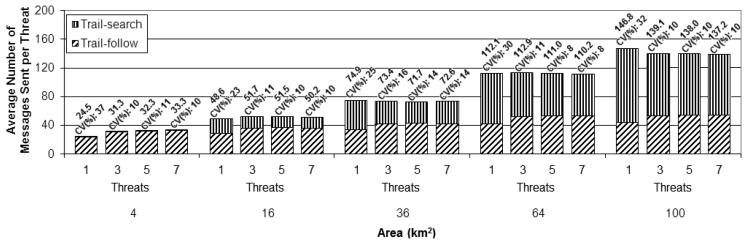
Results of the cost metric in terms of the average number of messages sent per threat for the simulations with one Unmanned Aerial Vehicle and the four different numbers of threats (1, 3, 5 and 7).

**Figure 12.**
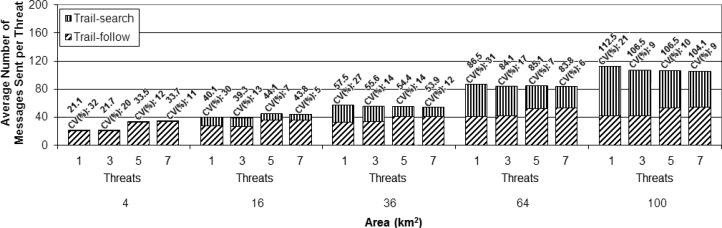
Results of the cost metric in terms of the average number of messages sent per threat for the simulations with 2 Unmanned Aerial Vehicles and the four different numbers of threats (1, 3, 5 and 7).

**Figure 13.**
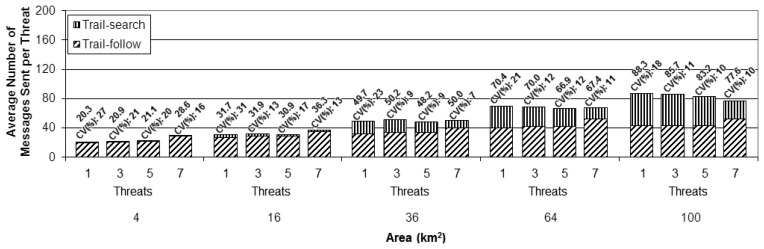
Results of the cost metric in terms of the average number of messages sent per threat for the simulations with 4 Unmanned Aerial Vehicles and the four different numbers of threats (1, 3, 5 and 7).

To perform a better analysis of the experimental results, the cost metric was normalized as the cost per threat instead of the total cost per setup, as presented in the previous simulation set. This normalization was done by dividing the total number of messages sent by the number of threats considered in each setup. This way of presenting the results makes the comparison of the different setups easier. Figures 11,12 and 13 present the results achieved for the cost metric per threat for the different numbers of UAVs. The upper part of the bars represents the contribution due to the trail-search mechanism, while the bottom part represents the contribution due to the trail-follow one. The numbers above the bars provide the average value and the coefficient of variance.

The following findings can be outlined:

1. From all charts in these figures it can be concluded that the cost associated with the trail-search mechanism is higher for the larger areas than for the smaller ones. This is due to the fact that the percentage of the area covered by trails with a given dimension is bigger in the smaller areas than it is in the larger ones.
2. For all numbers of threats and areas an increase in the number of UAVs implies a decrease in the cost associated to the trail-search. The reason for this phenomenon is that, with more UAVs, more trails are available in a given area, increasing the percentage of the area that is covered by them and also increasing the probability of an alarm to find a trail.
3. The cost associated with the trail-search mechanism turns out to be dependent of the number of threats. This is in contradiction with the intuitive observation that, as the results are presented per threat, the number of messages consumed by the trail-search mechanism should not increase with the number of threats. Observing each figure individually and comparing the results achieved for each number of threats, an interesting finding is made.

Analyzing the trail-search mechanism (upper part of the charts in Figures 11,12 and 13), in Figure 11 it is possible to observe a decrease in the cost metric due to this mechanism from setups with one threat to those with three threats, which is maintained while comparing setups with five to those with seven threats. In Figure 12, a similar behavior is noticed, but with a remarkable decrease from setups with three threats to those with five threats, which is maintained in setups with seven threats, and, finally, in Figure 13, a remarkable decrease from setups with five threats to those with seven threats can be observed. In the case of Figure 11 this denotes the situation when the setups with one UAV pass from the variation with just one threat to the others with more threats. In the case of Figure 12, the transition for the setups with two UAVs is from the variation with three to more threats, while in Figure 13 the transition for the setup with four UAVs is from the variation with five to the one with seven threats. This observed effect can be explained by the increased mobility that the increasing number of threats imposes in the different setups.

**Figure 14.**
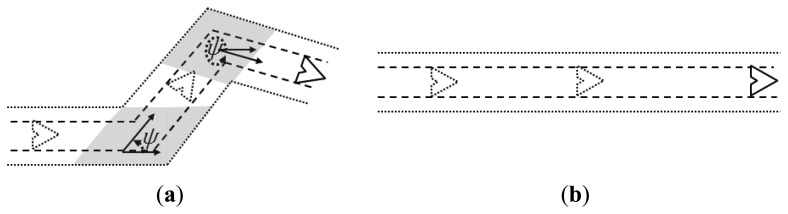
Area covered by a trail: (**a**) with overlap (parts colored in gray); (**b**) without overlap.

Considering the adopted movement model, the UAVs fly according to a random walk pattern, changing their heading angle after each movement step, as explained above. With the parameters used for the performed simulations, during a trail life time the UAVs may change their movement direction up to 10 times. This fact may create a number of pheromone overlapping areas in a trail, which as a result reduces the percentage of the total area that is effectively covered by a trail. Figure 14a depicts an example of trail with overlapping regions (in gray color), while Figure 14b shows another trail without overlap. Notice that for routes of the same size, overlapping regions are counted twice. This fact reduces the amount of sensor nodes covered by trails with overlapping regions compared to those without overlap.

When the UAVs receive an alarm and are requested to handle a threat, they perform a straight line movement from their current positions to the position in which the threat was detected. This movement is like the one presented in Figure 14b, thus creating trails without overlap and increasing the percentage of the area covered by a pheromone trail. In the setups with 1 UAV, for the variations with one threat the UAV will be performing its random movement, following a movement such as the one exemplified in Figure 14a. However, for the variations with more threats, after having received the first alarm the UAV will fly straight to the threat, as the example in Figure 14b, thus increasing the pheromone trail size, which increases the probability of an alarm finding a trail while performing a trail-search, thus reducing in average the number of messages used by this mechanism.

In many cases new alarms informing about other new threats will arrive within or close to the time window in which the UAV is already moving towards the most current threat to handle it. In this case, the trail will not present overlaps, or less overlaps compared to the trail left by a UAV performing the ordinary random-walk. For the setups with two and four UAVs a similar reasoning can be performed, but for a number of threats that exceeds the number of UAVs, which means that the UAVs will be receiving new alarms while going to handle or just after handling a previous one. In these cases, as they have moved in straight line, their trails have no or less overlaps and are then easier to find by the trail-search mechanism. Notice that for these two setups, with two and four UAVs, the number of threats immediately higher than the number of UAVs, three and five, respectively, do not stress much this observed behavior, but this becomes clearer for five and seven threats. This is explained by the fact that, with just one threat more than the number of UAVs, the average results do not change much, but when the difference is greater, the observed difference appears clearly, as it becomes more probable that the UAVs will have pending alarms in queue to be handled.

Analyzing the cost due to the trail-follow mechanism, it is possible to observe that the number of UAVs do not influence this mechanism. However, observing in each figure the variations according to the different numbers of threats, the same changing points observed and discussed for the trail-search mechanism results are perceived for each amount of UAVs. However, instead of a decrease in the number of messages sent, as it was observed in the trail-search mechanism results, we observe an increase in the number of messages sent due to the trail-follow mechanism. The reason for this fact is the same as explained above, *i.e.*, the increased size of the pheromone trail in the setup variations in which the number of threats exceeds the number of UAVs. With bigger trails, alarms forwarded by the trail-search mechanism can reach them in portions that are farther away from the UAVs, if compared to shorter trails, thus increasing the average of the obtained results of the trail-follow mechanism.

### 5.4. Study about the Influence of the Movement Pattern

From the analysis of the previous simulation set, a conclusion can be drawn in relation to the observed results: the movement pattern of the mobile nodes has a direct influence on the cost of the proposed pheromone-based alarm delivery. This statement is based on the observed differences in the results both of the trail-search and trail-follow mechanisms, when we consider the increase in the number of threats. This fact changes the movement pattern from the random-walk with a number of changes in the movement direction to a random-walk with longer straight line segments. This finding motivated the execution of a third simulation set. In this new set, the previous results using a random-walk for the UAVs' movement pattern with the above described parameters are compared to results achieved by a similar random-walk pattern, but with longer movement steps. The movement steps of this new set are of the size of the maximum length of the pheromone trails, *i.e.*, 5 km, instead of the movement step size originally used, *i.e.*, 500 m. This setup will prevent the UAVs from changing their direction while flying during the lifetime of a trail, unless they reach the edges of the surveillance area or in case they have to change their direction and move towards a threat just informed by a new alarm.

**Figure 15.**
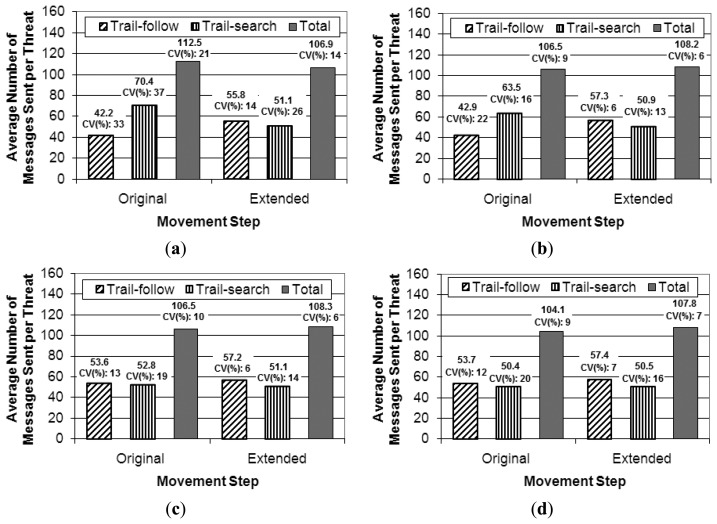
Results of the cost metric due to the trail-search and trail-follow mechanisms and to the total (trail-search plus trail-follow) for the simulations with 2 Unmanned Aerial Vehicles for both original and extended movement step: (**a**) one threat; (**b**) three threats; (**c**) five threats; (**d**) seven threats.

For this simulation set an area of 10 km × 10 km is used as scenario, in which two UAVs patrol the area and variations with the same number of threats were tested, *i.e.*, 1, 3, 5, and 7 threats. The assessed metric is the same as in the previous simulation set, namely the cost per threat for both parts of the pheromone-based approach, *i.e.*, trail-search and trail-follow mechanisms. We check whether the number of threats does not influence the cost results, thus confirming the conclusion about the influence due to the movement pattern. Figure 15 presents the results achieved for the extended movement step compared to the original one.

As can be observed in the results achieved with the extended movement step, the number of messages required by both parts of the coordination strategy is almost the same for the different number of threats, within a statistical variation. The point that presented the difference from the simulation variations from three to five threats observed with the original movement step is not observed in the results with the extended step (from Figure 15b to Figure 15c). This confirms the following: (a) the number of threats does not influence the results; and (b) in fact the observed differences in the previous simulation set are due to the change in the movement pattern that is created by the threat handling in the simulations with the original movement step. Moreover, a decrease in the values of the coefficient of variance can be noticed for both the trail-search and trail-follow mechanisms, which confirms that they became more deterministic in the results for the extended movement step than for the original step. For the total number of messages sent, there is no big difference between the results originally achieved when compared to the ones with the extended movement step. In relation to the variability of the results, Figure 15 shows that it is smaller with the extended step, as values of the coefficient of variance that comes together with the values of the averages are smaller for the extended step, which is consistent with the results for the trail-search and trail-follow mechanisms.

Even after concluding that the real effect of the different number of threats in the results is due to the difference that they imply in the UAVs movement pattern, we decided to keep the original movement step and the variation of the number of threats for the further simulation sets. This decision is based on the fact that simulation sets with varying step lengths would create an effect similar to varying the sizes of the areas with the same movement step, *i.e.*, varying the expected percentage of the area that can be covered by a trail, which was already tested.

Another aspect that is noteworthy to mention is that the study of different movement patterns is very dependent on specific application requirements. This is especially important for non-functional requirements, such as security and secrecy, due to the fact that some movement patterns can be more easily recognizable by hostile entities than others. In spite of the importance of this subject, a study about it is beyond the goals of this work. For further information about the influence of the movement patterns in routing algorithms for ad hoc networks, we refer to studies exclusively dedicated to this subject, such as [35,36].

### 5.5. Assessment of the Advantage of Using the Backbone in the Pheromone Trail

**Figure 16.**
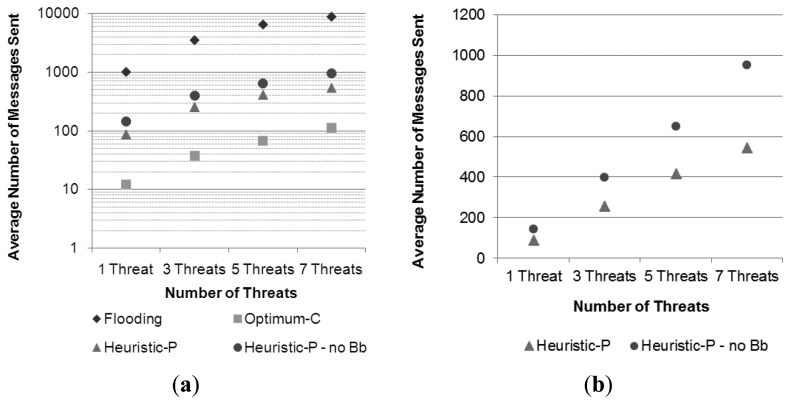
Cost of the pheromone strategy with (Heuristic-P) and without (Heuristic-P—no Bb) backbone: (**a**) cost comparison with the two reference solutions in a semi-log graph; (**b**) Cost comparison between the variations of the pheromone strategy in a linear graph.

Taking into account the distribution of pheromones over the ground sensor nodes presented in Section 4.3, and the proposal of using the central “backbone” part of the trail as a limiting border to the flooding of an alarm message that occurs inside a trail, a fourth set of simulations was performed to assess the effectiveness of this strategy. In this fourth set, the basic conditions presented in the first simulation sets were maintained, but the pheromone trail has no backbone, such that the alarm forwarding is performed as illustrated in Figure 7a. The performed simulation had a scenario with an area of 10 km × 10 km, in which four UAVs were patrolling the area, and there were the same increasing numbers of threats. Results for cost in terms of messages sent were compared to those of the corresponding setups presented in the previous experiments, where the pheromone trail has a backbone, as shown in Figure 16.

From the results in Figure 16a, which uses a semi-log scale, it is possible to compare both versions of the pheromone strategy with the reference solutions (flooding and optimal) and also to observe that they have a similar behavior, being much less costly than the flooding based one, and not too far from the optimal solution. A more detailed analysis between them is provided in Figure 16b, where a linear scale is adopted. The figure shows that the usage of the backbone, which was defined as having half of the width of the whole trail, provided a reduction of in average 30% in the number of messages sent for each number of threats. These results are consistent with those presented in Section 5.3, particularly the ones presented in Figure 13, which show that the trail-follow mechanism contributes with approximately 50% of the amount of messages required by the entire strategy, unless for the variation with seven threats, in which the trail-follow mechanism requires a higher percentage (Figure 13), as was discussed above. By using a backbone layer with half of the width of the trail, it is expected that the number of messages consumed by the trail-follow mechanism drops to half of the number of messages required by this mechanism in a trail without backbone.

As a result, the total number of messages sent by the entire pheromone strategy (trail-search and trail-follow) is expected to be reduced by in average 25%. Thus, considering the case for seven threats, in which the trail-follow mechanism requires relatively more messages, the achieved savings of approximately 30% is a reasonable result.

## 6. Conclusions

This paper presented a bio-inspired solution evoking the stigmergy concept to address the problem of routing messages from static sensor nodes to mobile ones in a hybrid wireless sensor network. This solution comprises a strategy to distribute information about the movement of the mobile sensor nodes over the static ones that mimics the spreading of pheromones by ants in their habitat. Results from simulations assessed how effective this approach is in diminishing the network traffic in relation to a conventional flooding based approach. The amounts of messages resulting from the proposed pheromone based approach were one order of magnitude lower than those required by flooding, *i.e.*, in the order of thousands for the flooding compared to hundreds for the proposed approach. This is a significant result as many approaches to solve the same problem rely on flooding based strategies to periodically update routing tables. Moreover, a scalability analysis is also presented, which shows an increase close to linear of the network traffic in relation to the increase in the network dimensions. This analysis allowed also a deeper understanding of the contributions of each part of the proposed pheromone strategy, *i.e.*, the trail-search and trail-follow mechanisms, as well as the movement model influence.

Further studies exploring different movement models are planned. Also, different combinations and variations of the simulation parameters can be tested to assess the efficiency of the approach in extreme conditions, such as narrowing the trail backbone, or testing a scenario in which the network is highly partitioned. Moreover, considering scenarios with mobile sensor nodes with different capabilities and different threats may represent an interesting variation, as the alarm messages can be intelligently driven to search for the most appropriate mobile sensor to handle a given threat.

## Figures and Tables

**Table 1.** Main simulation parameters and values.

**Parameters**	**Values**
Static Sensor Nodes Communication Range	350
Static Sensor Nodes Density	50 nodes/km^2^
UAV Altitude	250 m
UAV Movement Step	500 m
UAV Speed	100 km/h
UAV Communication Range	500 m
Pheromone Decay Rate	0.995 units/s

**Table 2.** Average, standard deviation and coefficient of variance for the number of messages sent using Heuristic-P, Optimum-C and Flooding in the first simulation set.

**Number of Threats**		**Heuristic-P**			**Optimum-C**			**Flooding**		

		**1 UAV**	**2 UAVs**	**4 UAVs**	**1 UAV**	**2 UAVs**	**4 UAVs**	**1 UAV**	**2 UAVs**	**4 UAVs**
**1**	**Avg.**	48.6	40.1	31.7	8.2	6.7	5.5	305.7	230.6	192.3
	**St. Dev.**	11.0	12.2	9.7	2.1	2.0	1.4	139.4	107.5	75.9
	**CV (%)**	23%	30%	31%	26%	30%	25%	46%	47%	39%
**3**	**Avg.**	155.0	118.0	95.5	23.1	20.5	18.5	691.0	566.0	391.9
	**St. Dev.**	24.0	20.0	15.7	5.2	3.8	3.0	277.1	213.5	122.4
	**CV (%)**	15%	17%	16%	23%	19%	16%	40%	38%	31%
**5**	**Avg.**	257.5	220.3	154.6	37.5	32.3	29.7	1,301.0	827.0	652.4
	**St. Dev.**	35.7	30.0	26.1	7.2	5.7	5.1	409.8	230.5	155.7
	**CV (%)**	14%	14%	17%	19%	18%	17%	31%	28%	24%
**7**	**Avg.**	315.1	306.6	254.3	54.7	49.1	37.6	1,715.4	1,561.7	1,285.8
	**St. Dev.**	45.1	40.1	34.5	8.3	7.6	6.7	519.1	427.7	326.8
	**CV (%)**	14%	13%	14%	15%	15%	18%	30%	27%	25%
